# Exercise: it's only a matter of time

**DOI:** 10.1113/JP280366

**Published:** 2020-08-04

**Authors:** Nicholas J. Saner, Matthew J‐C. Lee

**Affiliations:** ^1^ Sports Cardiology Baker Heart and Diabetes Institute Melbourne Australia; ^2^ Institute for Health and Sport (IHES) Victoria University Melbourne Australia

**Keywords:** circadian rhythm, clock genes, exercise

In today's modern society, approximately 20% of workers perform rotating shift work. Unfortunately, this poses considerably greater risks of developing cardio‐metabolic conditions, such as type 2 diabetes mellitus (T2DM). Indeed, periods of simulated shift work have been shown to directly compromise insulin sensitivity (Cedernaes *et al*. 2018), which has been attributed to factors including insufficient sleep quantity and quality, exposure to artificial light, and increased and/or mistimed food consumption. These factors are associated with the development of circadian misalignment – a condition whereby exogenous behavioural patterns (such as sleep–wake cycles or feeding schedules) do not align with endogenously generated circadian rhythms (biological rhythms that persist under constant conditions, over an approximate 24 h period (Gabriel & Zierath, 2019)). The link between circadian misalignment and T2DM is highlighted by the observation that forced experimental circadian desynchronisation can reduce insulin sensitivity (Cedernaes *et al*. 2018).

Circadian behaviours (such as sleep–wake cycles and activity patterns) are coordinated by the suprachiasmatic nucleus in the hypothalamus, which integrates environmental photic input and transduces this information to the peripheral tissues. At the molecular level, circadian rhythms are regulated by the autonomous activity of a core set of clock genes (namely *Bmal1*, *Clock*, *Per1/2 and Cry1/2*). Clock genes, and therefore circadian rhythms, are influenced by environmental time cues known as zeitgebers (literally ‘time givers’); common examples include light exposure and timing of food consumption. The recently published work by Kemler *et al*. (2020) now provides clear evidence that exercise can also be considered a potent zeitgeber. Using both *in vivo* and *in vitro* models, they eloquently demonstrate that exercise elicits specific time‐of‐day effects on circadian rhythms and the core molecular clock machinery in skeletal muscle. These findings provide a foundation for further investigations into the complex interplay between circadian rhythms, metabolic health, and exercise, from which time‐appropriate, therapeutic strategies could be developed to benefit those most at risk of circadian misalignment, such as shift workers and ageing populations.

In the initial *in vivo* experiment, PERIOD2::LUCIFERASE (PER2::LUC) mice were used to examine the effects of exercise timing on circadian rhythmicity. In these mice, luciferase reporter genes were inserted into the *Per2* gene, so that the subsequent PER2 protein expression also contained luciferase activity; thus, the detected changes in bioluminescence represent changes in PER2 protein levels. This technique allows changes in the core clock machinery, and the circadian clock phase, to be constantly measured over several days. The mice performed a single 60 min treadmill run at one of three times of the day: the middle of the resting (light) phase (ZT5), the end of the resting (light) phase (ZT11), and in the middle of the active (dark) phase (ZT17). Exercising at ZT5 caused PER2 protein content to peak approximately 100 min earlier (i.e. a phase advance). Exercising at ZT11 delayed peak PER2 protein expression by approximately 62 min, and there was no phase shift when exercising at ZT17. While previous studies have shown that exercise *training* can influence the circadian phase, Kemler *et al*. (2020) are the first to report that a *single exercise session* can shift the circadian phase in a direction that is dependent on when the exercise is conducted. Given that several populations, including a significant portion of the workforce, are routinely exposed to risk factors for circadian misalignment, this work provides important insights that will enable further exploration into the potential therapeutic effects of exercise on circadian rhythmicity. It has been speculated that exercise interventions incorporating specifically timed sessions might be useful for realigning circadian rhythms (Gabriel & Zierath, 2019). For example, scheduling an exercise session to either advance or delay a molecular marker of a particular circadian phase might confer therapeutic effects to those experiencing circadian misalignment, such as shift workers.

A true environmental time cue (i.e. a zeitgeber) interacts with the core molecular clock to shift an endogenous rhythm. Exercise promotes many systemic and local physiological responses that may also influence circadian rhythms (e.g. changes in temperature, hormone concentrations, and tissue‐specific transcription and translation). Therefore, before exercise (or specifically, muscle contraction) could be considered a true zeitgeber, it was necessary to confirm its influence within an isolated environment (e.g. in cell culture). Kemler *et al*. (2020) generated C2C12 myotubes that exhibited an endogenous rhythm of core clock gene expression, and contained a BMAL1::Luciferase reporter gene. At times associated with the highest, mid, and lowest levels of *Bmal1* mRNA expression, the myotubes were electrically stimulated with pulse contractions designed to replicate the contractions experienced by mice during treadmill running (25 ms pulse, 10 V at 6 Hz, every 5 s). As with their previous experiment, the myotube contractions also elicited specific time‐of‐day‐dependent effects. Compared with unstimulated control cells, the oscillatory bioluminescence pattern occurred earlier when exercise was performed at mid *Bmal1* mRNA expression, whereas significant phase delays were observed when contractions were performed at the peak or trough of *Bmal1* mRNA expression. By removing the influence of other systemic effects of exercise, these results suggest that muscle contraction *per se* can be considered a true zeitgeber.

The phase delay observed when myotubes were electrically stimulated at time points corresponding to both the highest and lowest points of *Bmal1* mRNA expression was also evident when the PER2::LUC mice exercised at the end of the resting phase (ZT11), when *Bmal1* mRNA expression was lowest. However, the mice were not also exercised when *Bmal1* mRNA expression peaked; therefore, we can only speculate from the data in myotubes that a similar effect would have occurred. While many circadian functions are known to be conserved between mice and humans, a vital next step is to investigate how these findings could be applied to humans. van Moorsel *et al*. (2016) demonstrated that human skeletal muscle *Bmal1* mRNA expression, as well as mitochondrial respiratory function and whole‐body resting energy expenditure exhibit daily fluctuations that peak at approximately 23:00 h. Furthermore, Cedernaes *et al*. (2018) demonstrated that one night of simulated shift work disrupts *Bmal1* mRNA expression and concomitantly decreases glucose tolerance. Therefore, it would be intriguing to examine whether exercising at specific times of the day (corresponding to the daily undulations in *Bmal1* mRNA expression) could realign the molecular clock, and therefore protect against the myriad detrimental metabolic effects associated with circadian misalignment. Furthermore, if changes in core clock gene expression can be associated with changes in other, more easily assessed circadian parameters (e.g. melatonin levels or core body temperature), this may facilitate the prescription of specifically timed exercise interventions, individualised to the busy routines imposed by our 24 h lifestyles.

Kemler *et al*. (2020) also examined the time‐of‐day effects of myotube contractions on the expression of core clock genes. When contractions were performed at mid *Bmal1* mRNA expression, *Per2* mRNA expression significantly decreased. When performed at both the peak and trough of *Bmal1* mRNA expression, the contractions induced divergent changes in clock gene expression (despite having similarly delayed the BMAL1::LUC bioluminescence rhythm in the previous experiment). Compared with the control cells, there was a significant decrease in *Bmal1*, *Per1* and *Per2* mRNA expression when contractions were performed at the trough of *Bmal1* expression, whereas no changes were observed when performed at the peak of *Bmal1* expression. These results suggest that different components of the molecular clock are differentially regulated according to when exercise is performed. While only core clock gene expression was studied, the authors’ *in vitro* model lends itself to further investigations into the effects of exercise timing on additional downstream effectors, particularly the gene expression and protein content of other metabolic targets regulated by clock genes. The content of many metabolically relevant proteins (e.g. PGC‐1α, GLUT4, TBC1D1 and PDH kinase) are reduced in clock gene knockout animals, often with concomitant reductions in glucose tolerance and mitochondrial content and/or function. Therefore, alterations in clock gene expression, now understood to be influenced by exercise timing, may have implications for the activity of metabolic regulatory proteins, and glucose tolerance; these measures should be incorporated into future studies employing this *in vitro* system. Such investigations would prove useful in elucidating the mechanistic basis of recent reports demonstrating that short‐term high‐intensity interval training (HIIT) performed in the afternoon was more effective at improving the glycaemic control of men with type 2 diabetes than HIIT in the morning (Savikj *et al*. 2019). Furthermore, in mouse skeletal muscle, Sato *et al*. (2019) demonstrated that exercise performed at different times of the day elicits distinct metabolic and transcriptomic signatures. Specifically, exercising early in the active phase led to significantly greater changes in in the total number of metabolites and transcripts compared with exercising early in the rest phase. Together, these findings clearly demonstrate time‐of‐day‐dependent effects of exercise and further support the concept that precisely timed exercise sessions may help to maximise several health benefits (Gabriel & Zierath, 2019). Despite these preliminary findings, more work is needed to fully elucidate these effects.

In summary, Kemler *et al*. (2020) have demonstrated that exercise has a time‐of‐day‐specific influence on circadian rhythms and is a zeitgeber for skeletal muscle. The methods employed in this study can be further utilised to investigate the underlying mechanisms for time‐of‐day‐specific, exercise‐induced health benefits, and improve our current understanding of the link between exercise and circadian rhythms. The potential therapeutic uses of exercise to realign circadian rhythms or to alter the core molecular clock in humans requires considerable research, but may present a useful strategy for mitigating the detrimental metabolic effects of circadian misalignment that affect several populations.

## Additional information

### Competing interests

None.

### Author contributions

All authors have approved the final version of the manuscript and agree to be accountable for all aspects of the work. All persons designated as authors qualify for authorship and all those who qualify for authorship are listed.

### Funding

None.
